# Supplementary material for: Imaging analysis of six human histone H1 variants reveals universal enrichment of H1.2, H1.3, and H1.5 at the nuclear periphery and nucleolar H1X presence
Source: eLife. 2024 Mar 26;12:RP91306. doi: 10.7554/eLife.91306 (PMC10965224; doi:10.7554/eLife.91306)

A

Blots left panel (T47D, MCF-7, HeLa, HepG2, SK-N-SH)

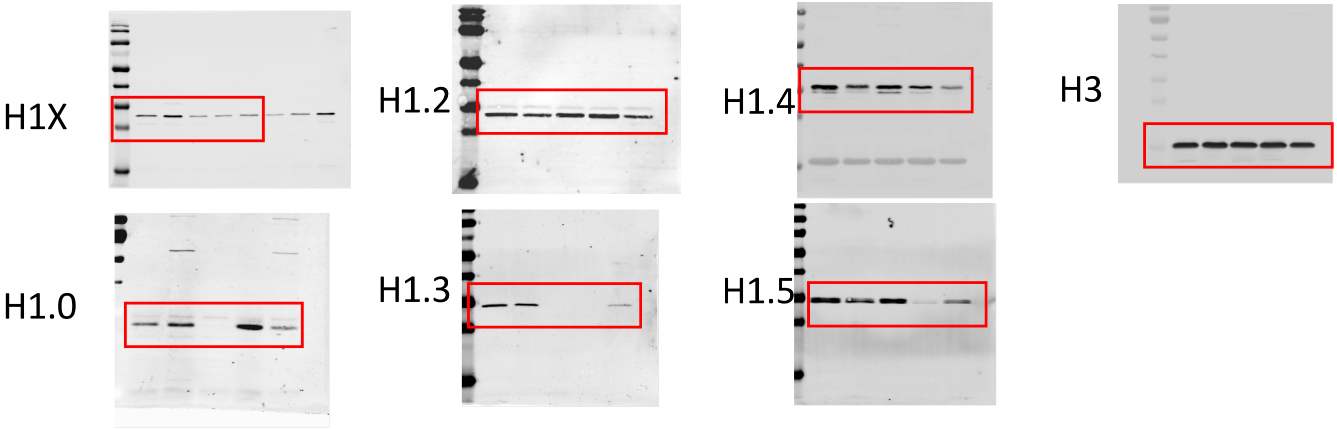

Blots right panel (CaCo2, HCT-116, HT-29, 293T, NT2-D1)

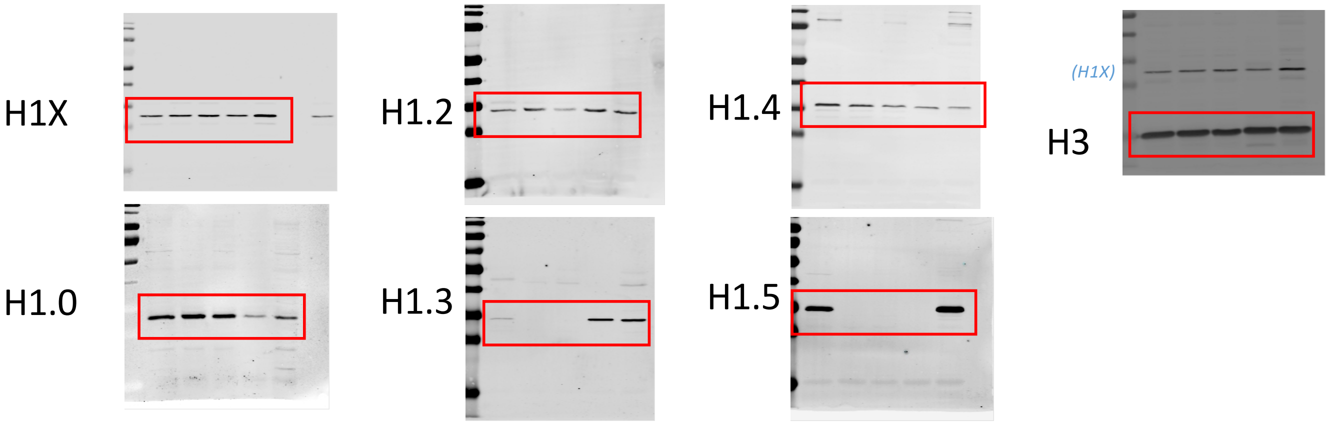

C

Blots left panel (SK-MEL-147, SK-MEL-173, UACC-257)

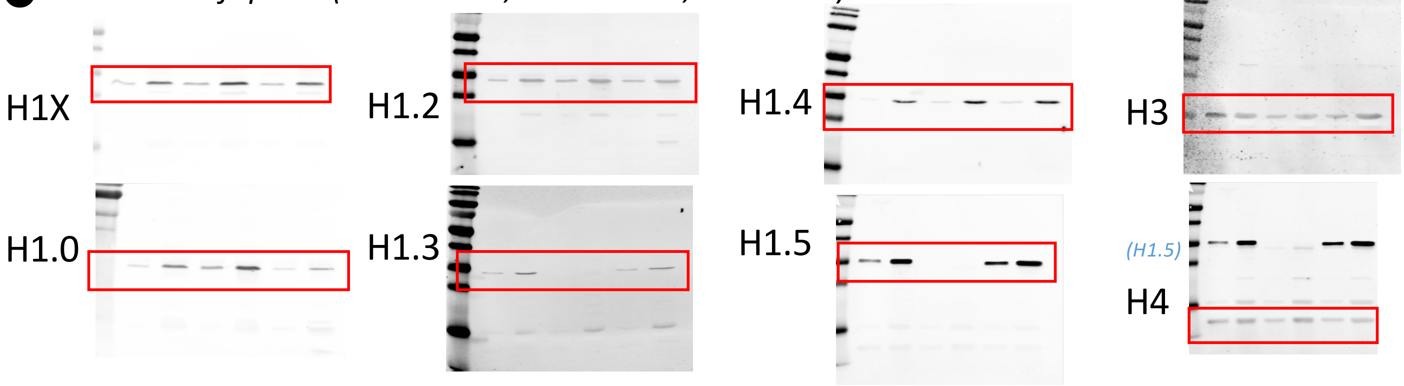

Blots right panel (IGR-39, SK-MEL-28, WM266.4)

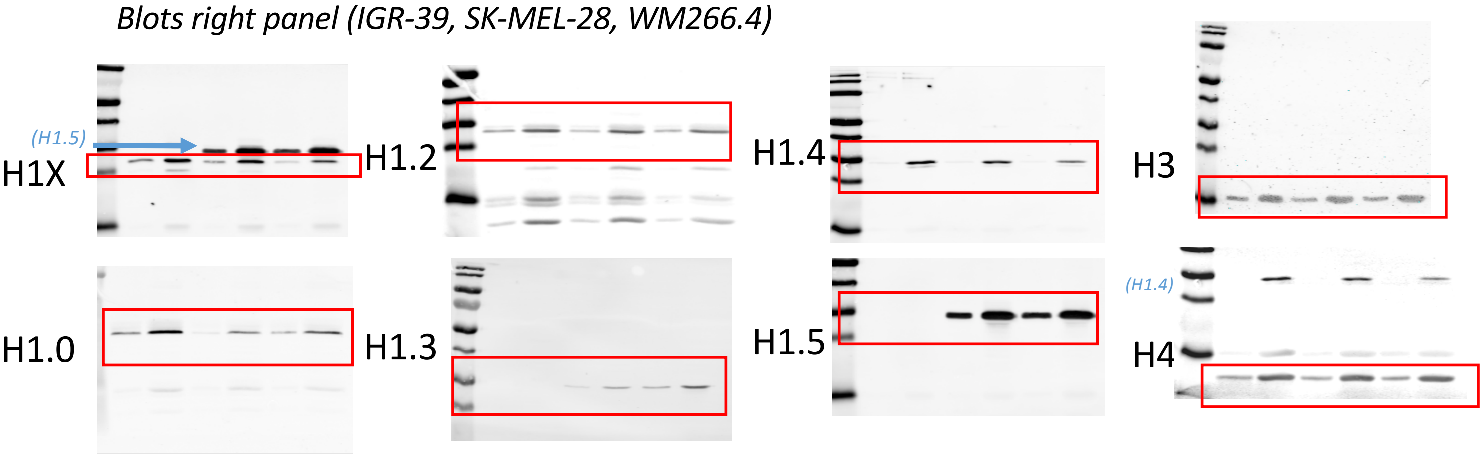

Supplement: Figure 5—figure supplement 1—source data 1. [file elife-91306-fig5-figsupp1-data1.pdf]
