# Supplementary figures and images for: Imaging analysis of six human histone H1 variants reveals universal enrichment of H1.2, H1.3, and H1.5 at the nuclear periphery and nucleolar H1X presence
Source: eLife. 2024 Mar 26;12:RP91306. doi: 10.7554/eLife.91306 (PMC10965224; doi:10.7554/eLife.91306)

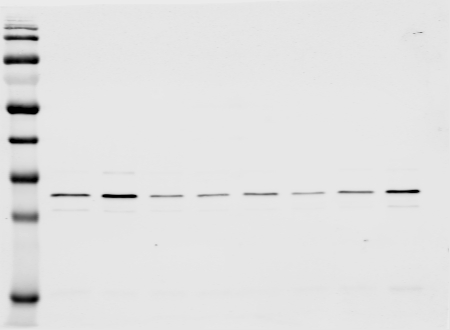

Supplement: Figure 5—figure supplement 1—source data 2. [file elife-91306-fig5-figsupp1-data2.jpg]

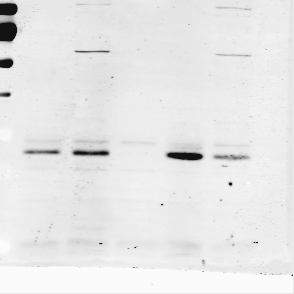

Supplement: Figure 5—figure supplement 1—source data 3. [file elife-91306-fig5-figsupp1-data3.jpg]

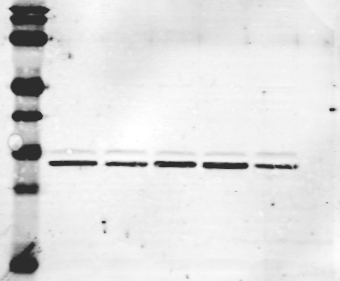

Supplement: Figure 5—figure supplement 1—source data 4. [file elife-91306-fig5-figsupp1-data4.jpg]

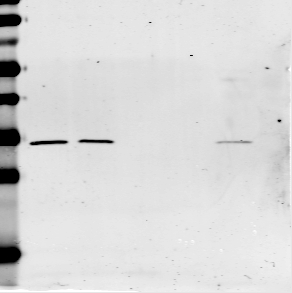

Supplement: Figure 5—figure supplement 1—source data 5. [file elife-91306-fig5-figsupp1-data5.jpg]

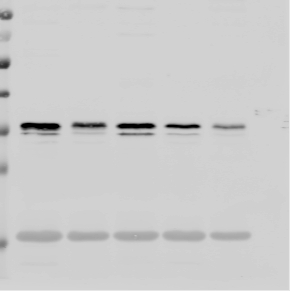

Supplement: Figure 5—figure supplement 1—source data 6. [file elife-91306-fig5-figsupp1-data6.jpg]

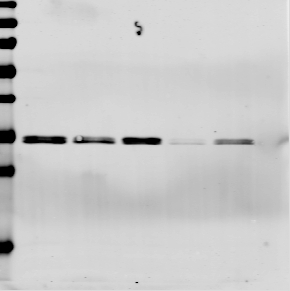

Supplement: Figure 5—figure supplement 1—source data 7. [file elife-91306-fig5-figsupp1-data7.jpg]

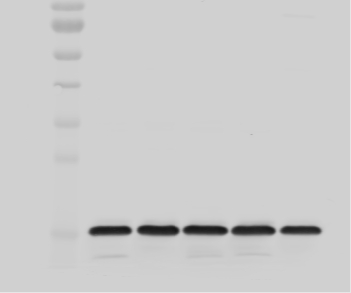

Supplement: Figure 5—figure supplement 1—source data 8. [file elife-91306-fig5-figsupp1-data8.jpg]

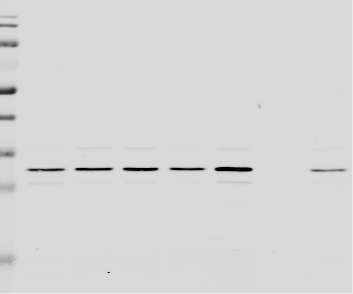

Supplement: Figure 5—figure supplement 1—source data 9. [file elife-91306-fig5-figsupp1-data9.jpg]

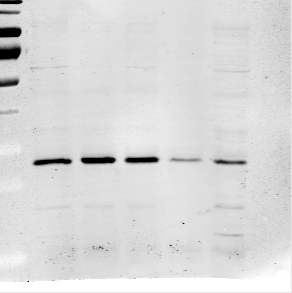

Supplement: Figure 5—figure supplement 1—source data 10. [file elife-91306-fig5-figsupp1-data10.jpg]

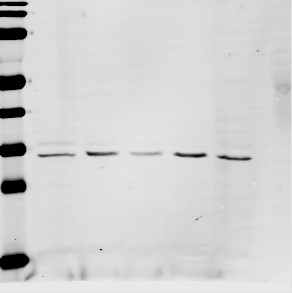

Supplement: Figure 5—figure supplement 1—source data 11. [file elife-91306-fig5-figsupp1-data11.jpg]

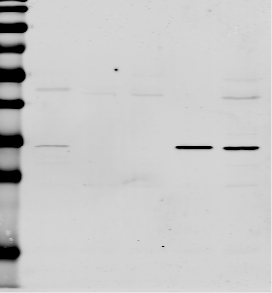

Supplement: Figure 5—figure supplement 1—source data 12. [file elife-91306-fig5-figsupp1-data12.jpg]

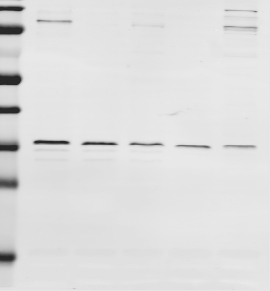

Supplement: Figure 5—figure supplement 1—source data 13. [file elife-91306-fig5-figsupp1-data13.jpg]

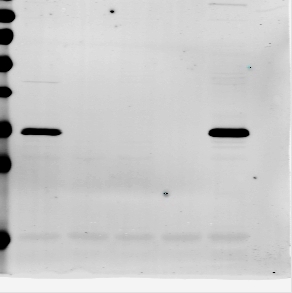

Supplement: Figure 5—figure supplement 1—source data 14. [file elife-91306-fig5-figsupp1-data14.jpg]

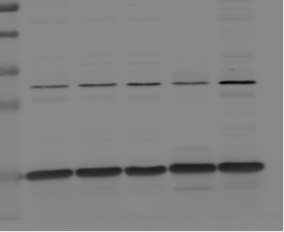

Supplement: Figure 5—figure supplement 1—source data 15. [file elife-91306-fig5-figsupp1-data15.jpg]

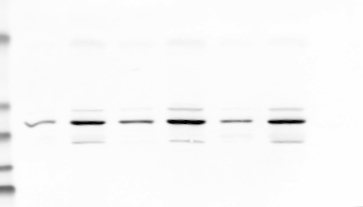

Supplement: Figure 5—figure supplement 1—source data 16. [file elife-91306-fig5-figsupp1-data16.tif]

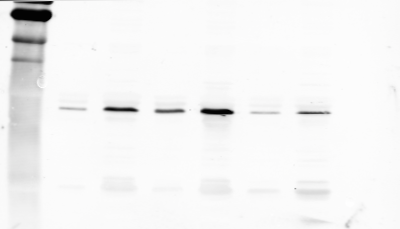

Supplement: Figure 5—figure supplement 1—source data 17. [file elife-91306-fig5-figsupp1-data17.tif]

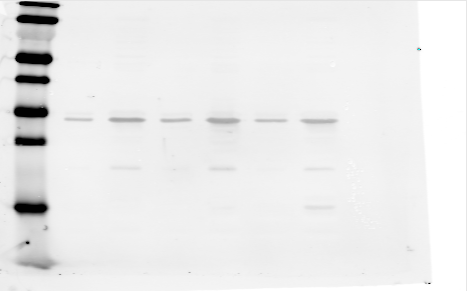

Supplement: Figure 5—figure supplement 1—source data 18. [file elife-91306-fig5-figsupp1-data18.tif]

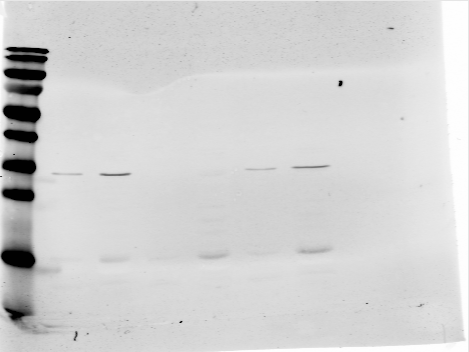

Supplement: Figure 5—figure supplement 1—source data 19. [file elife-91306-fig5-figsupp1-data19.tif]

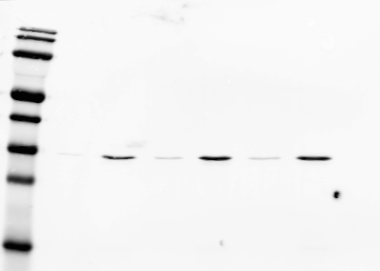

Supplement: Figure 5—figure supplement 1—source data 20. [file elife-91306-fig5-figsupp1-data20.tif]

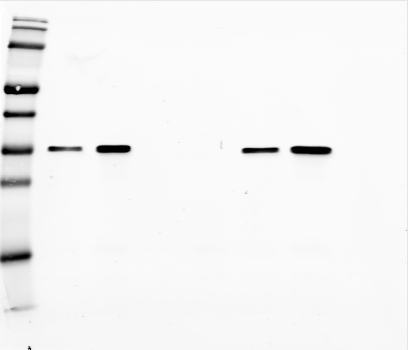

Supplement: Figure 5—figure supplement 1—source data 21. [file elife-91306-fig5-figsupp1-data21.tif]

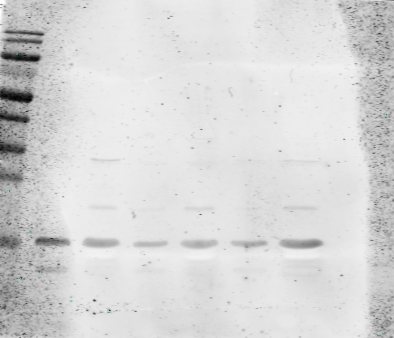

Supplement: Figure 5—figure supplement 1—source data 22. [file elife-91306-fig5-figsupp1-data22.tif]

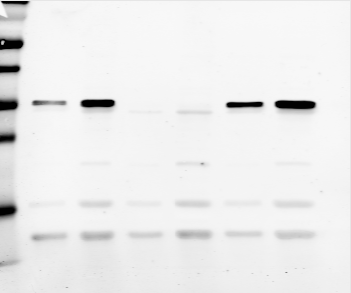

Supplement: Figure 5—figure supplement 1—source data 23. [file elife-91306-fig5-figsupp1-data23.tif]

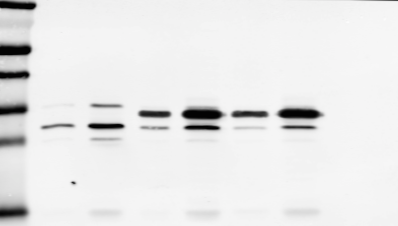

Supplement: Figure 5—figure supplement 1—source data 24. [file elife-91306-fig5-figsupp1-data24.tif]

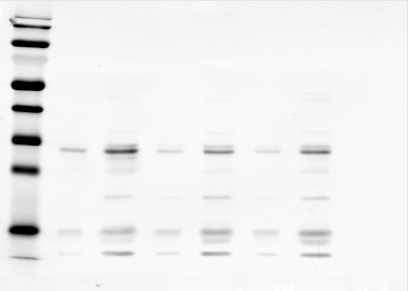

Supplement: Figure 5—figure supplement 1—source data 26. [file elife-91306-fig5-figsupp1-data26.tif]

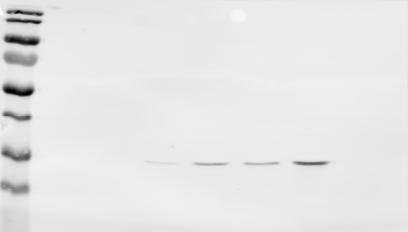

Supplement: Figure 5—figure supplement 1—source data 27. [file elife-91306-fig5-figsupp1-data27.tif]

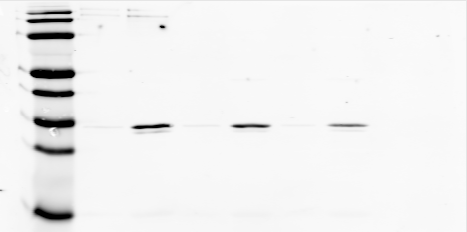

Supplement: Figure 5—figure supplement 1—source data 28. [file elife-91306-fig5-figsupp1-data28.tif]

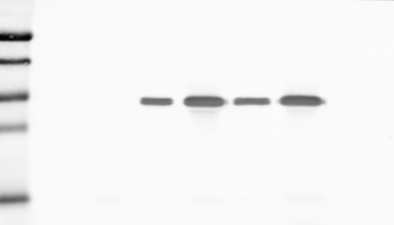

Supplement: Figure 5—figure supplement 1—source data 29. [file elife-91306-fig5-figsupp1-data29.tif]

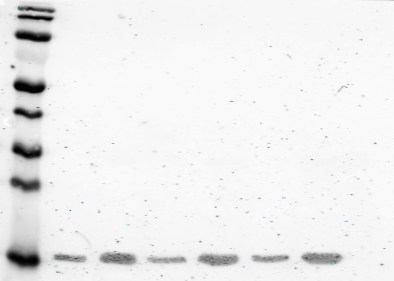

Supplement: Figure 5—figure supplement 1—source data 30. [file elife-91306-fig5-figsupp1-data30.tif]

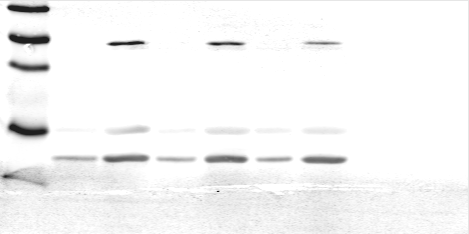

Supplement: Figure 5—figure supplement 1—source data 31. [file elife-91306-fig5-figsupp1-data31.tif]
