# Supplementary material for: Imaging analysis of six human histone H1 variants reveals universal enrichment of H1.2, H1.3, and H1.5 at the nuclear periphery and nucleolar H1X presence
Source: eLife. 2024 Mar 26;12:RP91306. doi: 10.7554/eLife.91306 (PMC10965224; doi:10.7554/eLife.91306)
Supplement: Supplementary file 1. — Forward (F) and reverse (R) oligonucleotides for the indicated genes are shown. [file elife-91306-supp1.docx]

**Supplementary File 1. Oligonucleotides for semiquantitative PCR.** Forward (F) and reverse (R) oligonucleotides for the indicated genes are shown.

|  | **SENSE** | **SEQUENCE (from 5' to 3')** |
| --- | --- | --- |
| **H1.0** | F | CCTGCGGCCAAGCCCAAGCG |
|  | R | AACTTGATCTGCGAGTCAGC |
| **H1.1** | F | CTCCTCTAAGGAGCGTGGTG |
|  | R | GAGGACGCCTTCTTGTTGAG |
| **H1.2** | F | GGCTGGGGGTACGCCT |
|  | R | TTAGGTTTGGTTCCGCCC |
| **H1.3** | F | CTGCTCCACTTGCTCCTACC |
|  | R | GCAAGCGCTTTCTTAAGC |
| **H1.4** | F | GTCGGGTTCCTTCAAACTCA |
|  | R | CTTCTTCGCCTTCTTTGGG |
| **H1.5** | F | CATTAAGCTGGGCCTCAAGA |
|  | R | TCACTGCCTTTTTCGCCCC |
| **H1X** | F | CCCAACGATGTAGCGTTTTT |
|  | R | AAGGCCGAGAGCCAATAGA |
| **IFi27** | F | TGCTCTCACCTCATCAGCAGT |
|  | R | CACAACTCCTCCAATCACAACT |
| **OASL** | F | GGGACAGAGATGGCACTGAT |
|  | R | AAATGCTCCTGCCTCAGAAA |
| **IFIT2** | F | ACGGTATGCTTGGAACGATTG |
|  | R | AACCCAGAGTGTGGCTGATG |
| **IFIT3** | F | CGGAACAGCAGAGACACAGA |
|  | R | ATGGCATTTCAGCTGTGGA |
| **DDX60** | F | AAGGTGTTCCTTGATGATCTCC |
|  | R | TGACAATGGGAGTTGATATTCC |
| **IFi6** | F | CTGTGCCCATCTATCAGCAG |
|  | R | GGGCTCCGTCACTAGACCTT |
| **SST1** | F | AACCACTGTGACGGGAGAAA |
|  | R | CTGGGACAGGACGAGACAC |
| **SATa** | F | AAGGTCAATGGCAGAAAAGAA |
|  | R | CAACGAAGGCCACAAGATGTC |
| **HERVK** | F | AGAGGAAGGAATGCCTCTTGCAG |
|  | R | TTACAAAGCAGTATTGCTGCCCGC |
